# The optimal WC cut-off points for the prediction of subclinical CVD as measured by carotid intima-media thickness among African adults: a cross-sectional study

**DOI:** 10.1186/s12872-021-02389-5

**Published:** 2021-12-01

**Authors:** M. C. Ringane, S. S. R. Choma

**Affiliations:** Department of Pathology and Medical Sciences, University of Limpopo, Private Bag X1106, Sovenga, South Africa

**Keywords:** Cardiovascular diseases, Waist circumference, Carotid intima-media thickness, Obesity, Visceral obesity, Cut-off points

## Abstract

**Background:**

Increased waist circumference (WC) is one of the cardiovascular disease (CVD) risk factors used to predict cardiovascular events. Waist circumference cut-off values for predicting metabolic syndrome and other cardiovascular risks have been previously studied. Carotid intima-media thickness (CIMT) is one of the cardiovascular risk factor recently described and reported to be suitable as it is a direct measurement of vascular quality. Hence the aim of the present study was to determine the optimal WC cut-off point for the prediction of subclinical CVD.

**Methods:**

The study was a cross-sectional study using quantitative methods, conducted among 1318 adults aged between 40 and 60 years old, residing in a rural Black population in Limpopo province. Carotid Intima-Media Thickness measurements were performed using a LOGIQ ultrasound system (GE Healthcare, CT, USA). Waist Circumference (WC) (cm) was measured to the nearest 0.1 cm. Bivariate correlation, logistic regression and receiver operating characteristic were analysed using the statistical package for social sciences version 26.0 software.

**Results:**

Among the total population, 69% were women and 31% men with a mean age of 53 ± 7 years. Among women, WC at a cut-off value of 95 cm gave the highest sensitivity of 57%, the specificity of 55% and an area under the curve (AUC) of 0.588. In men, an optimum WC cut-off point of 82 cm yielded the highest sensitivity and specificity at 72% and 70% respectively, with an AUC of 0.767 *p* < 0.001.

**Conclusion:**

The traditional waist circumference cut-off points (94 cm for women and 80 cm for men) that are currently used for the diagnosis of metabolic syndrome might not be suitable in the prediction of an increased CIMT.

## Introduction

The prevalence of cardiovascular diseases (CVDs) and their associated risk factors are reported to be increasing worldwide [1]. In 2015, CVDs were responsible for about 17.9 million deaths worldwide [1, 2]. Previously, a high prevalence of CVD risks was seen in developed countries, but recent data confirms that half of the global burden of CVDs were reported among low and middle-income countries [3]. This might be due to epidemiological transition, urbanisation, economic development, adoption of a western lifestyle and the improvement of living conditions [4–6]. Or it could be due to the increase in the prevalence of obesity, the major risk factor for CVD [4]. In Africa, 38% of all non-communicable disease-related deaths were attributed to CVD [7] and 1 million deaths in sub-Saharan Africa were linked to CVD, contributing 5.5% of the global CVD related deaths [7].

The risk for the development of CVD can be assessed at the subclinical stage [8] by direct assessment of vascular changes, and/or indirectly by assessing the presence of the vascular risk factors [9]. The direct assessment methods include computerised tomography (CT), Magnetic resonance angiography (MRA) and ultrasound scanning [10]. Ultrasound is usually used for the measurements of the carotid intima-media thickness (CIMT) and the presence of plaque for the assessment of atherosclerosis [11]. Ultrasound is the preferred method for epidemiological studies as it is less complicated, inexpensive and non-invasive as it does not expose one to radiation [12].

Obesity, particularly abdominal obesity, is associated with several cardiometabolic risk factors and is also independently associated with atherosclerotic CVDs [13, 14]. Several reports have documented a positive association between abdominal obesity and CIMT [15–17]. Abdominal obesity can be assessed using different techniques, among all the techniques waist circumference (WC) is the most commonly used technique as it is non-invasive, cheap and easy to perform compared to Magnetic Resonance Imaging (MRI), computed tomography (CT) and ultrasound [18, 19].

There are several WC cut-off points used to define the risk for CVD. The International Diabetes Federation (IDF) recommended the cut-off point for abdominal obesity using a WC of 94 cm in men and 80 cm in women for European and African descended population [20], while the National Cholesterol Educational Programme Adult Treatment Panel III (NCEP-ATP III) recommends the use of 102 cm for men and 88 cm for women as the cut-off points for defining abdominal obesity without regard to race [21]. However, these standardised cut-offs were concluded based on the European population and they are being used by researchers among sub-Saharan Africans [20]. This might lead to the misdiagnosis of abdominal obesity among Africans since there are discrepancies in fat distribution across different populations [22].

Several studies have tried to come up with waist circumference cut off values among Africans [23–26], these studies used metabolic syndrome as an outcome event. Recently, CIMT has been recognised as one of the better measure of a subclinical atherosclerosis a CVD risk factor [11]. Therefore, it is of a vital importance to go beyond the use of only traditional CVD risk factors, as the absence and/or presence of the risk factors does not excludes the presence and/or absence of a vascular disease [27]. Hence, the risk for CVD can be better predicted by directly assessing the vascular changes through CIMT. The use of CIMT has been supported by The Screening for Heart Attack Prevention and Education (SHAPE) Task Force [28] and Society of Atherosclerosis Imaging and Prevention [29].

It will be interesting to determine which waist circumference cut-off values could be used to predict a better outcome event (CIMT), as opposed to metabolic syndrome. According to our knowledge, there has not been any study that determined the waist circumference cut off values to predict subclinical CVD among Africans. The aim of the present study was therefore to determine an optimal WC cut-off for the prediction of subclinical CVD as measured by Carotid Intima-Media Thickness among African adults.

## Methods

### Study design and population

The study was a cross-sectional study using quantitative methods. The study was conducted among 1318 adults, aged between 40 and 60 years old, residing in a rural population of Limpopo, DIMAMO formerly called Dikgale Health and Demographic surveillance system (HDSS) centre. Dikgale is a rural area occupied by Black South Africans predominantly Northern Sotho (Sepedi) speaking. This rural area is composed of 15 rural villages with a total population of 35,000. DIMAMO is situated in South Africa, Limpopo Province, Capricorn district, approximately 30 km away from the University of Limpopo and about 40–50 km northeast of Polokwane, the capital city of Limpopo Province.

### Sample collection

For analysis of possible CVD risk factors, overnight fasting blood was collected by a professional nurse from each participant into EDTA-coated tubes (purple top tubes) for blood lipids, and sodium fluoride/potassium oxalate coated tubes (grey top tubes) for glucose analysis. The tubes were centrifuged at 3000 rpm for 10 min and the resulted plasma was separated from red cells within 6 h of collection and transferred into 2 ml cryotubes for storage. The samples were transported to the University of Limpopo laboratory (chemical pathology laboratory) for storage and analysis. Plasma was stored at − 80 °C until use. Glucose tests were performed immediately after separation.

### Data collection

Participants with a history of CVD or its treatment, pregnant women and those with incomplete data were excluded from study participation. Anthropometric measurements performed were weight, height and WC. Weight (kg) was measured to the nearest 0.1 kg with the respondent wearing light clothes and without shoes using the Omron-B100 scale. Height (m) was measured to the nearest 0.1 m using a stadiometer. Participants were requested to stand vertically on the stadiometer without shoes. Body mass index (BMI) was calculated dividing weight in kilograms by height measured in meters squared. Waist circumference (WC) (cm) was measured to the nearest 0.1 cm at the level of the iliac crest while the participant was at minimal respiration.

Carotid Intima-Media Thickness measurements were done using a LOGIQe ultrasound system (GE Healthcare, CT, USA), according to detailed procedures and methods that were previously published by Ali et al., 2018. For CVD risk factors, Blood pressure was measured using a digital sphygmomanometer (Omron M6, Omron, Kyoto, Japan), detailed procedure [30]; fasting blood glucose and lipids were determined using the AU480 auto analyzer from Beckman Coulter.

### Outcome

In both genders, a CIMT of ≥ 0.80 mm was classified as an increased CIMT [31].

### Predictors

In this study, CVD risk factors assessed were obesity, abdominal obesity measured by WC, increased CIMT, hypertension, diabetes and dyslipidaemia. Obesity was defined by a BMI ≥ 30 kg/m^2^ [32]. Abdominal obesity was defined by a WC of ≥ 94 cm in men and 80 cm in women [20].

### Covariates

Hypertension was defined as a resting SBP ≥ 140 mmHg and/or DBP ≥ 90 mmHg [33]. Diabetes was defined as a fasting blood glucose of ≥ 7.0 mmol/L [34]. Dyslipidaemia was defined as one or more of the following: Triglyceride ≥ 1.7 mmol/L, Total Cholesterol ≥ 5.2 mmol/L, High-Density Lipoprotein-Cholesterol (< 1.03 mmol/L in men, < 1.29 mmol/L in women) and Low-Density Lipoprotein-Cholesterol ≥ 3.4 mmol/L [21].

### Data analysis

Data were analysed using the Statistical Package for Social Sciences (SPSS) version 26.0 software. The distributions of variables were determined using skewness and kurtosis test, variables that were normally distributed were expressed as mean $\pm$ standard deviation. In the present study, the following tests were used: An Independent Student t-test was to compare the means of CIMT, biochemical and anthropometric measurements between men and women. Chi-square was used to compare the prevalence of cardiometabolic risk factors. Bivariate correlation was used to determine the association between WC and CIMT. Partial correlation was used to determine the association between WC and CIMT after controlling for age and gender. Linear regression was used to determine whether WC was a predictor of an increased CIMT. The receiver operating characteristics (ROC) curve was used to determine an optimal WC cut-off in predicting an increased CIMT. The optimal cut-off values of waist circumference were calculated by plotting the true-positive rate (sensitivity) against the false-positive rate (1-specificity). For all analysis, statistical significance was set at a probability (*p*) level of 0.05.

## Results

Baseline characteristics of the study population are summarised in Table 1. The study was conducted among 1318 participants from a rural population of which 69% were women and 31% were men. The mean age was 52 ± 8 years and there was no significant difference between men and women. The overall mean BMI, WC and CIMT were 27.7 ± 8.0 kg/m^2^, 89.6 ± 16.0 cm and 0.65 ± 0.12 mm, respectively. Women had a higher mean BMI, WC, DBP, LDL-c and glucose compared to men (*p* < 0.001), while CIMT and other biochemical measurements were not significantly different across gender. The overall prevalence of obesity (BMI ≥ 30 km/m^2^), abdominal obesity (WC ≥ 94 cm in men and ≥ 80 cm in women) and an increased CIMT were 35%, 58% and 9.5% respectively. The prevalence of obesity and abdominal obesity was higher among women than men (*p* < 0.001). There was no significant difference in the prevalence of an increased CIMT between genders (*p* = 0.301).

**Table 1:** Baseline characteristics of the study population by gender

Variables	Total population	Gender		*p* Value
		Women	Men	
N	1318	910(69%)	408(31%)	
Age (years)	52 ± 8	52 ± 8.00	51 ± 8.32	0.699
BMI (Kg/m^2^)	27.73 ± 8.00	30.44 ± 7.84	21.70 ± 4.05	≤ 0.001**
Overweight %(N)	307(23.3%)	236(25.9%)	71(17%)	0.142
Obese %(N)	461(35.0%)	448(49.2%)	13(3.2%)	≤ 0.001**
Overweight/Obese %(N)	65%(166)	85.5%(141)	27.2%(25)	0.001**
WC(cm)	89.60 ± 16.03	93.67 ± 16.07	80.36 ± 11.59	≤ 0.001**
High WC %(N)	766(58.1%)	709(77.9%)	57 (14%)	0.001**
Right CIMT (mm)	0.65 ± 0.14	0.65 ± 0.14	0.65 ± 0.14	0.946
Left CIMT (mm)	0.64 ± 0.13	0.63 ± 0.13	0.64 ± 0.13	0.339
CIMT (mm)	0.65 ± 0.12	0.64 ± 0.12	0.65 ± 0.13	0.587
High CIMT %(N)	129 (9.7%)	86 (9.5%)	43(10.5%)	0.301
SBP (mmHg)	126 ± 22.00	126 ± 22	126 ± 21	0.741
DBP (mmHg)	81 ± 13.00	81 ± 13	79. ± 13	≤ 0.001**
Hypertensive %(N)	33%(85)	40.0%(66)	20.7%(19)	0.001**
TRIG (mmol/L)	1.11 ± 0.62	1.11 ± 0.60	1.10 ± 0.65	0.875
High TRIG %(N)	14%(37)	14.9%(24)	14.4%(13)	0.539
LDLc (mmol/L)	2.41 ± 0.93	2.53 ± 0.94	2.16 ± 0.84	≤ 0.001**
High LDL c %(N)	29%(74)	31.5%(52)	24.2%(22)	0.136
HDLc (mmol/L)	1.21 ± 0.40	1.18 ± 0.36	1.26 ± 0.48	0.001**
Low HDL c %(N)	68%(175)	77.6%(128)	51.1%(47)	0.001**
CHOL (mmol/L)	4.12 ± 1.10	4.21 ± 1.12	3.93 ± 1.00	≤ 0.001**
High CHOL %(N)	35%(90)	37.0%(61)	31.5%(29)	0.230
Glucose (mmol/L)	5.22 ± 2.24	5.31 ± 2.44	5.04 ± 2.10	0.054*
High glucose %(N)	11%(27)	9.1%(15)	13.0%(12)	0.216

*Significant at *p* value ≤ 0.05, **Significant at *p* value ≤ 0.001

Bivariate correlation shows a positive relationship between WC with both sides of CIMT measurements and their average (*p* =  ≤ 0.001). Waist circumference and CIMT remained significantly associated even after controlling for gender and age (*p* =  ≤ 0.001). Among other measurements of obesity, WC was found to be the only predictor of an increased CIMT (*p* = 0.007). Individuals with high WC were found to be 1.78 times more likely to be risk for an increased CIMT. See Table 2. Hypertension and glucose were positively associated with an increased CIMT (*p* =  ≤ 0.001), while BMI, insulin, cholesterol, triglyceride, LDL-C and dyslipidaemia were not associated with CIMT. Linear regression analysis was again performed across gender. Among women, age, WC, hypertension and glucose were positively associated with an increased CIMT (*p* = 0.001, 0.006, 0.001 and 0.003). While insulin was negatively associated with an increased CIMT (*p* = 0.025). Dyslipidaemia, cholesterol, triglyceride, LDL-C and BMI were not associated with an increased CIMT (Table 3). Among men, age, WC, hypertension and glucose were positively associated with an increased CIMT (*p* = 0.001). While insulin, dyslipidaemia cholesterol, triglyceride, LDL-C and BMI were not associated with an increased CIMT (Table 4).

**Table 2:** Backward linear regression for assessment of the association of WC with an increased CIMT

Variables	CIMT 1st model		CIMT last model	
	Odds ratio	*p* Value	Odds ratio	*p* Value
Age	0.385	≤ 0.001**	0.380	≤ 0.001**
BMI	0.998	0.997		
WC	1.76	0.032*	1.78	0.007*
Hypertension	1.99	0.005*	1.97	0.005*
Glucose	1.96	0.003*	1.99	0.001**
Insulin	0.45	0.194		
Cholesterol	1.01	0.984		
Triglyceride	1.22	0.471		
LDL-C	0.947	0.890		
Dyslipidaemia	0.901	0.652		
R2	0.405		0.546	
LR chi2(4)	59.20		37.86	
Prob > chi2	≤ 0.001		≤ 0.001	
Pseudo R2	0.0583		0.0448	
Log likelihood	− 397.67413		− 403.34468	
Likelihood-ratio test	LR chi2(1)	21.34		
	Prob > chi2	0.00159		

*Significant at *p* value ≤ 0.05, **Significant at *p* value ≤ 0.001

**Table 3:** Backward linear regression for assessment of the association of WC with an increased CIMT women

Variables	CIMT 1st model		CIMT last model	
	Coef	*p* Value	Coeff	*p* Value
Age	0.385	≤ 0.001**	0.380	≤ 0.001**
BMI	0.998	0.997		
WC	0.160	0.032*	0.169	0.006*
Hypertension	0.099	0.005*	0.137	≤ 0.001**
Glucose	0.102	0.003*	0.120	0.003*
Insulin	− 0.453	0.025	− 0.330	0.035*
Cholesterol	0.014	0.764		
Triglyceride	0.022	0.371		
LDL-C	0.045	0.569		
Dyslipidaemia	0.019	0.681		
R2	0.400		0.512	
LR chi2(4)	54.10		35.16	
Prob > chi2	≤ 0.001		≤ 0.001	
Pseudo R2	0.0583		0.0448	
Log likelihood	−396.35413		− 408.26468	
Likelihood-ratio test	LR chi2(1)	18.94		
	Prob > chi2	0.00197		

*Significant at *p* value ≤ 0.05, **Significant at *p* value ≤ 0.001

**Table 4:** Backward linear regression for assessment of the association of WC with an increased CIMT men

Variables	CIMT 1st model		CIMT last model	
	Coef	*p* Value	Coeff	*p* Value
Age	0.450	≤ 0.001**	0.444	≤ 0.001**
BMI	0.034	0.733		
WC	0.216	≤ 0.032*	0.265	≤ 0.001**
Hypertension	0.118	≤ 0.005*	0.117	0.005*
Glucose	0.128	≤ 0.003*	0.136	≤ 0.001**
Insulin	0.045	0.135		
Cholesterol	0.114	0.523		
Triglyceride	0.035	0.241		
LDL-C	0.137	0.149		
Dyslipidaemia	0.031	0.454		
R2	0.473		0.534	
LR chi2(4)	56.13		38.23	
Prob > chi2	≤ 0.001		≤ 0.001	
Pseudo R2	0.0613		0.0540	
Log likelihood	− 390.41345		− 405.68264	
Likelihood-ratio test	LR chi2(1)	17.90		
	Prob > chi2	0.00648		

*Significant at *p* value ≤ 0.05, **Significant at *p* value ≤ 0.001

**Fig. 1:**
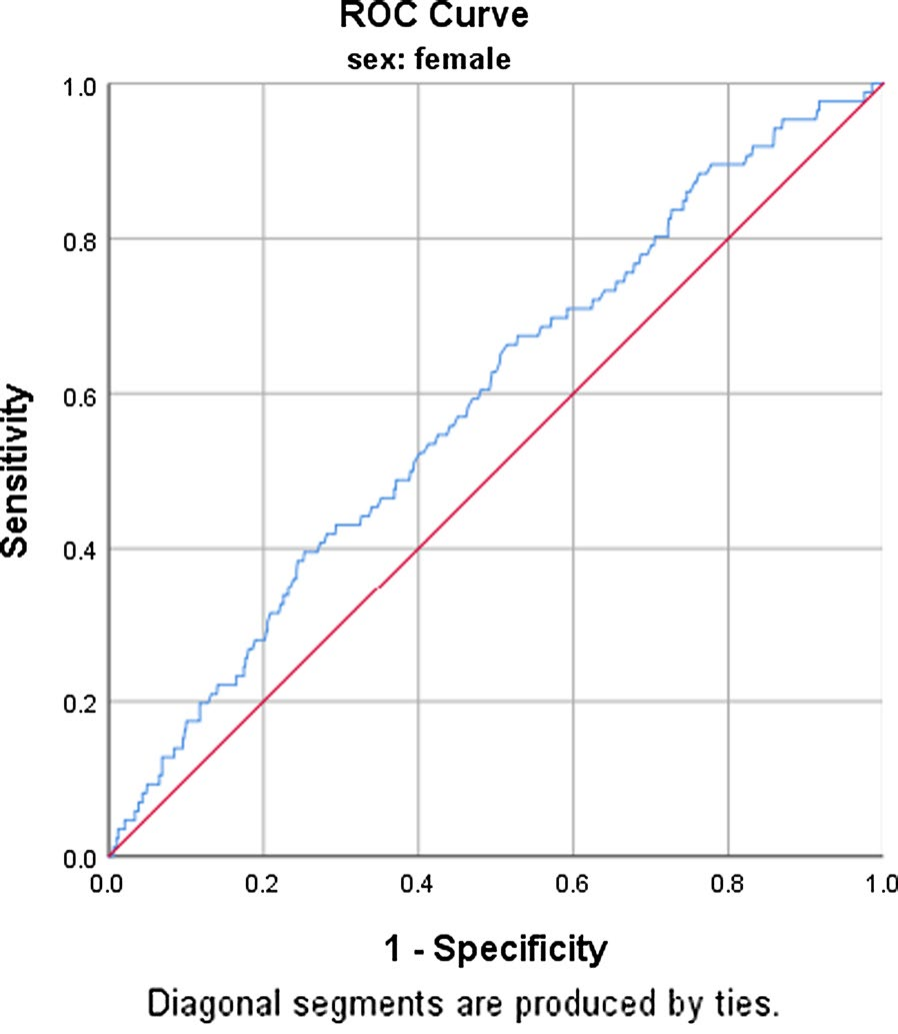
Receiver Operating Characteristics (ROC) curve for waist circumference to predict the risk of an increased CIMT (> 0.80 mm) in women

**Fig. 2:**
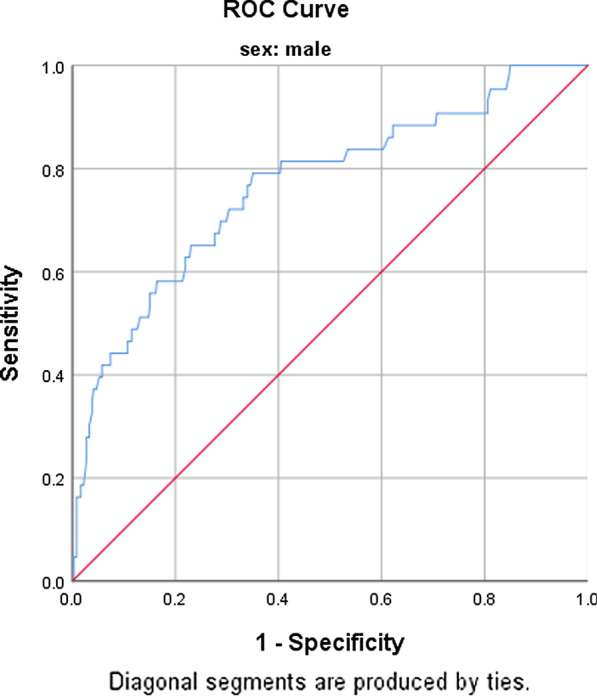
ROC curve for waist circumference as a predictor of an increased CIMT (> 0.80 mm) in men

The receiver operating characteristics (ROC) curve and its coordinates are summarised in Figs. 1 and 2 as well as Table 5, respectively. Among women, WC at a cut-off value of 95 cm gave the highest sensitivity of 57%, the specificity of 55% and an area under the curve (AUC) of 0.588. The Youden index (YI), 95% confidence interval (CI), positive likelihood ratio (PLR), positive predictive value (PPV) and the *p* value were 0.120, 0.526–0.650, 1.3, 56% and *p* = 0.007, respectively. When using sensitivity, a cut-off point of 90 cm yielded the highest sensitivity of 70%. Specificity, PLR, PPV and YI of 43%, 1.2, 55% and 1.2 respectively. For men, WC at a cut-off point of 82 cm yielded the highest sensitivity of 72%, a specificity of 70% with its corresponding AUC of 0.767, 95% CI (0.685–0.848), PLR (2.4) and a *p* value of *p* ≤ 0.001.

**Table 5:** Coordinates of the curve

	CIMT								
	OCP (cm)	SEN (%)	SPE (%)	AUC	95% CI	YI	PLR	PPV%	*p* value
	Female								
WC	95	57	55	0.588	0.526–0.650	0.12	1.3	56	0.007*
	90	70	43	0.588	0.526–0.650	0.13	1.2	55	
	*Male*								
WC	82	72	70	0.767	0.685–0.848	0.417	2.4	71	≤ 0.001**

*OCP* optimum cut-off point, *SEN* sensitivity, *SPE* specificity, *AUC* area under curve, *YI* Youden index, *CI* confidence interval

*Significant at *p* value ≤ 0.05, **Significant at *p* value ≤ 0.001

Table 6 summarises WC cut-off points previously reported among African countries as well as from the present study. Several studies investigated optimal WC cut-off points for the prediction of CVD risk among African populations. These studies used different CVD risk factors as an outcome factor and also reported different optimal WC cut-off points for each risk factor. Using CIMT as an outcome, the present study reported a WC cut-off point of ≥ 82 cm for men and ≥ 95 cm for women. This was different to the cut-off points reported by several South African studies using Metabolic Syndrome [23–25, 35] and to those recommended by WHO and IDF.

**Table 6:** Comparison of the optimal cut-off point of waist circumference (WC) for the diagnosis of metabolic syndrome in African countries with the present study

Author	Country	Participants	Outcome variable	Cut-off point for men (cm)	Cut-off point for women (cm)
Motala et al. [23]	South Africa	189	MS	≥ 86	≥ 92
Crowther et al. [24]	South Africa	1251	MS	–	≥ 91
Hoebel et al. [25]	South Africa	152	MS	≥ 92	≥ 94
Owolabi et al. [35]	South Africa	998	MS	≥ 95	≥ 90
Murphy et al. [26]	Uganda	6136	MS	≥ 78 to ≥ 80	≥ 82 to ≥ 85
Present study	South Africa	1318	CIMT	≥ 82	≥ 95

## Discussion

In the present study, a high prevalence of selected cardiovascular risk factors was observed. Among the study population, 35% were obese and 58% had a high waist circumference. This was higher compared to 23.6% and 34% reported in the same province by Sengwayo et al. [36] and Maimela et al. [37], respectively. The difference between these two findings might be due to the different WC cut-off points used. In the present study IDF cut-off points of ≥ 94 cm for men and ≥ 80 cm for women were used, while Maimela’s study used the NCEP cut off points of ≥ 102 cm for men and ≥ 88 cm for women. Women had a higher BMI and waist circumference than men. This was also observed in studies conducted in the same population [36–39]. This might be due to a lack of physical activity among women as compared to men [37].

The prevalence of an increased CIMT was 9.7%. This is low compared to the prevalence observed in a study conducted among Black South Africans by Holland et al. [31]. This may be because the present study is a population-based study that used apparently healthy participants whilst Holland et al. [31] study used participants with established coronary artery disease. In the present study, CIMT did not show any significant difference between men and women. This was supported by a population-based study conducted in Nigeria [40].

Hypertension, one of the risk factors for CVD, was observed among 33% of the study population. This finding was comparable to several studies conducted in the Black rural population [38, 41, 42]. In the present study, women were seen to be more hypertensive as compared to men. The same pattern was reported in previous studies conducted in the same area by Peltzer & Phaswana-mafuya [43] and Alberts et al. [38]. The prevalence of Diabetes (using American Diabetes Association criteria for fasting glucose of ≥ 7.0 mmol/L) was found to be 11%. This is in line with the prevalence reported in KwaZulu-Natal [44] and the same areas as in the present study [38].

In the present study, the prevalence of most cardiovascular risk factors was higher compared to previous studies conducted among the African population. This confirms a continuous increase in the prevalence of CVD risk factors reported by Teo and Dokainish [3].

Previously, CVD risk was only assessed by the presence of CVD risk factors such as old age, hypertension, obesity, high cholesterol and low HDL [45]. There is some experimental evidence against the use of only CVD risk factors in identifying individuals at high risk from the normal ones [46, 47]. It was reported that traditional CVD risk factors alone explain only 60–65% of the CVD risk, and the presence of one or more risk factors is common even among individuals who may not develop a clinical disease [48]. Some acute clinical events may occur among patients without any CVD risk factors [48]. As a result, the better approach currently proposed is to use both CVD risk factors and the information obtained from the assessment of vascular changes of the carotid arteries using an ultrasound [12]. It is reported that the measurement of CIMT provides more accurate information as compared to conventional risk factors, hence it is regarded as a valuable method of assessing CVD risk factors [40].

Previous studies have reported a positive association between cardiovascular outcomes with an increased CIMT [49–51]. An increased CIMT was also positively associated with presence of conventional CVD risk factors [16, 52, 53]. Abdominal obesity measured through WC was reported to be one of the traditional risk factors for CVDs [17]. In the present study, WC was found to be positively associated with CIMT and also a predictor of an increased CIMT. These findings have also been reported in previous studies [15, 54].

According to the authors’ knowledge, the present study is the first to determine the optimal WC cut-off points for predicting an increased CIMT as the authors did not find any study that has assessed the optimal WC cut-off points where an increased CIMT was the outcome event. It is also the first study of this kind conducted among Africans. As a result, the findings of the present study are mostly compared with studies investigating WC cut-off points where the outcome event is either metabolic syndrome or other cardiometabolic risk factors.

There are several studies investigated the optimum WC cut-off points in both rural and urban parts of SA and Africa (Table 6), but the outcome variable were other cardiovascular risk factors [23–26, 35], not an increased CIMT. There are inconsistencies regarding the optimal WC cut-off points for predicting different cardiovascular risk factors across the globe, among African countries and within South Africa (Table 6). Since CIMT is gaining recognition as an indicator of an early progression of atherosclerosis and a risk factor for CVD, it is important to establish the optimal cut-off points for waist circumference using CIMT as an output variable.

As indicated earlier this is the first study to determine the WC cut off values for predicting CIMT and thus there are no available studies to compare to. Hence, findings from the present study were compared to studies used Metabolic syndrome as an outcome event. In the present study, a cut-off point of 95 cm for women yielded the highest AUC, sensitivity and specificity at 0.588, 57% and 55%, respectively. Regardless of the statistical significance of the cut-off (*p* = 0.007), the sensitivity, specificity and AUC are slightly low to justify the use of this cut-off. This was low due to, both sensitivity and specificity were used to identify an optimum cut-off. When sensitivity is used, a cut-off point of 90 cm yielded the highest sensitivity of 70% and a specificity of 43%. This was also low when compared to AUC, sensitivity and specificity (0.713, 88% and 46% respectively) yielded by a cut-off point of 89.46 cm reported by Owolabi et al. [35] and also when compared to AUC, sensitivity and specificity observed among men. Since specificity and sensitivity of a diagnostic test can differ with the disease prevalence [55], low AUC, specificity and sensitivity among women might be due to differences in sample spectrum between women and men. Another study conducted in SA, reported a cut-off of 92 cm among women (sensitivity 45.9% and specificity 81.9%) [25]. In this study, specificity was prioritised over sensitivity.

For men, a cut-off point of 82 cm yielded the highest AUC, sensitivity and specificity of 0.767, 72% and 70% respectively (*p* = 0.001). This was low compared to the WC cut-off point reported by several studies here in SA (Table 4) [23–26, 35] and 94 cm recommended by IDF. Unlike for women, the sensitivity, AUC and specificity produced by a cut-off for were justifiable high to consider the use of this cut-off. At the traditional WC cut-off value of 94 cm, the sensitivity and specificity dropped to 59.3% and 52.3% respectively. Inconsistency regarding the WC cut-off has been observed between the present study and other studies conducted among African population (Table 6). This was also observed even among the studies that used the same outcome (metabolic syndrome).

To the author’s acknowledge, the present study is the first of its kind and as a result data supporting the present study’s findings is limited. The second limitation is that the present study is conducted among one race residing in a rural area and probably one ethnic group and thus findings of the present study may not apply among other population groups.

## Conclusion

From the results of the present study, it is concluded that the prevalence of CVD and its associated risk factors are continuing to increase among Black rural populations. Both traditional risk factors and the measurement of CIMT should be used to assess CVD risk. The conventional waist circumference cut-off points (94 cm for men and 80 cm for women) that are being used for predicting metabolic syndrome or other conventional risk factors may not be appropriate for predicting an increased CIMT. Supported by findings from the present and previous studies, a WC cut-off specifically to each risk factor should be generated and used other than using the same cut-off for all risk factors. The present study thus suggests that the waist circumference of 90 cm for women and 82 cm for men is suitable for predicting increased CIMT among this population.

## Limitations

To the author’s acknowledge, the present study is the first of its kind and as a result data supporting the present study’s findings is limited. The second limitation is that the present study is conducted among one race residing in a rural area and probably one ethnic group and thus findings of the present study may not apply among other population groups.

## Recommendations

Similar studies should be conducted among different ethnic groups and races to validate the results from the present study.

## Data Availability

The datasets used and/or analysed during the current study are available from the corresponding authors on reasonable request.

## Abbreviations

CIMT: Carotid intima-media thickness
CI: Confidence interval
WC: Waist circumference
DBP: Diastolic blood pressure
SBP: Systolic blood pressure
BMI: Body mass index
CVD: Cardiovascular disease
ROC: Receiver operating characteristics
YI: Youden index
AUC: Area under the curve
PLR: Positive likelihood ratio
WHO: World Health Organisation
IDF: International Diabetes Federation
NCEP-ATP III: National cholesterol education-adult treatment panel III
HDL-c: High-density lipoprotein-cholesterol
LDL-c: Low-density lipoprotein-cholesterol
CT: Computerised tomography
